# Supplementary figures and images for: Adeno-Associated Virus 2 (AAV2) - Induced RPA exhaustion generates cellular DNA damage and restricts viral gene expression
Source: PLoS Pathog. 2025 Aug 18;21(8):e1013142. doi: 10.1371/journal.ppat.1013142 (PMC12373274; doi:10.1371/journal.ppat.1013142)

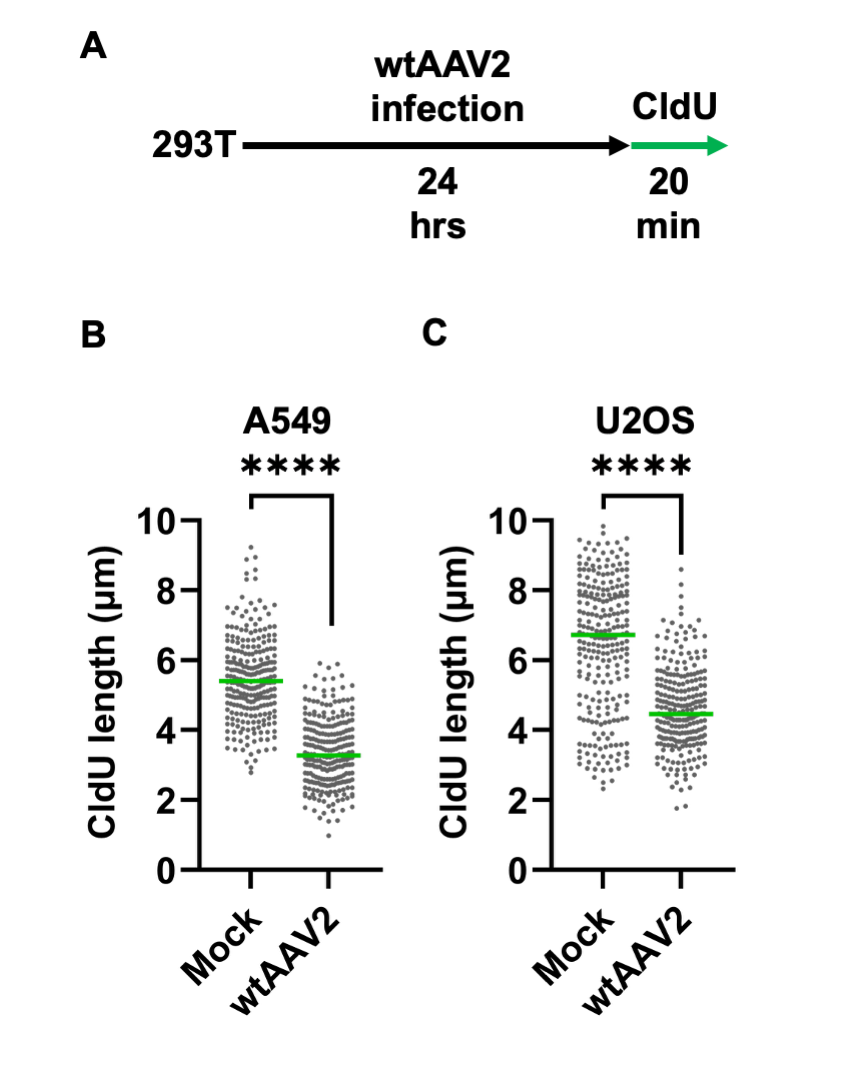

Supplement: S1 Fig — (A) Schematic of DNA fiber analysis using CldU pulsing of (B) A549 and (C) U2OS cells at 24 hpi when infected at an MOI of 5,000 vg/cell. Data is represented as median of 2 independent experiments with at least 100 datapoints per sample per replicate. Statistical analysis was performed using Mann Whitney Wilcoxon test with p value being depicted by ****, p < 0.0001. (TIFF) [file ppat.1013142.s001.tiff]

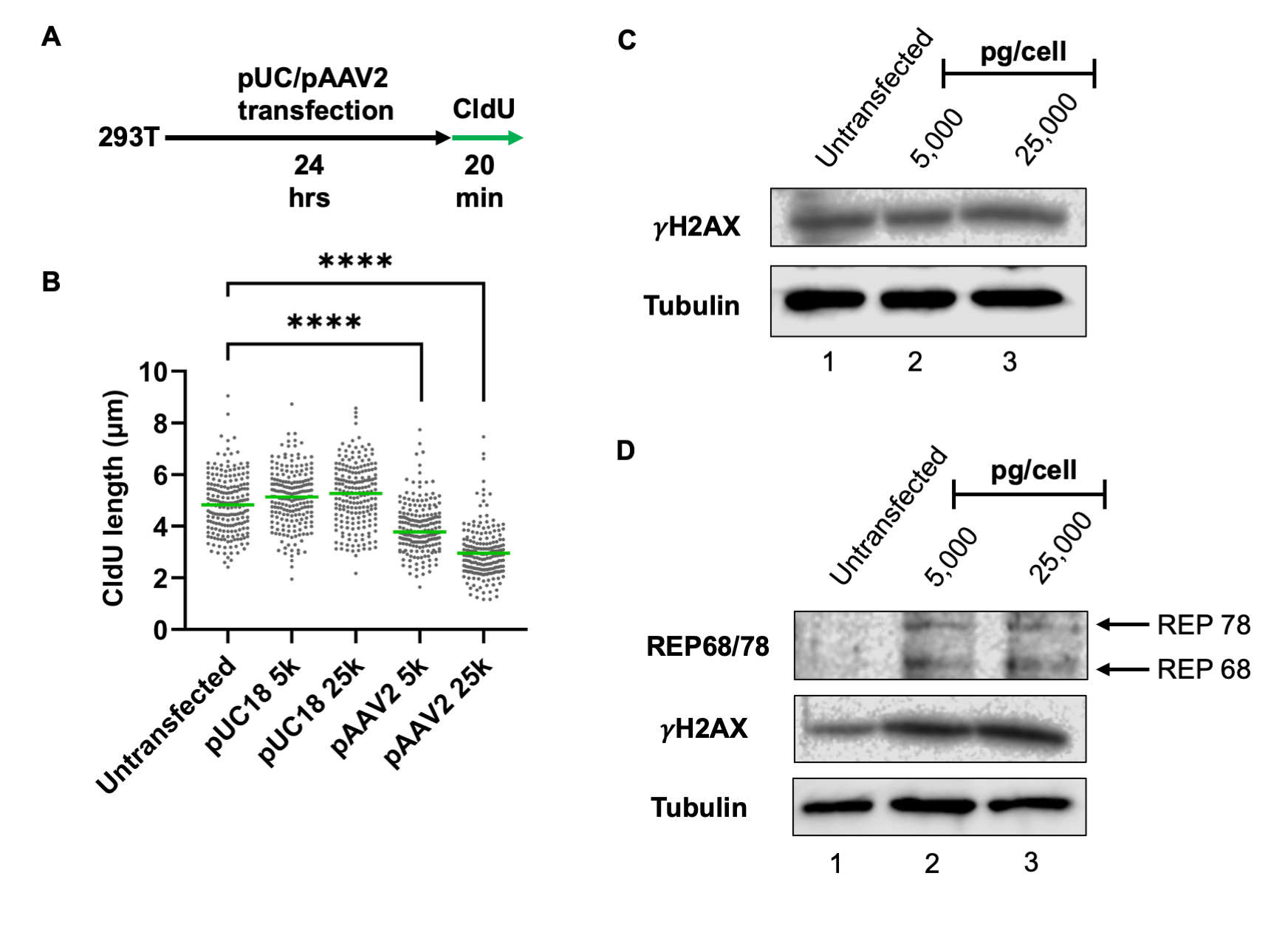

Supplement: S2 Fig — (A) Schematic of pUC18/pAAV2 transfection of 293T cells followed by DNA fiber analysis at 24 hpi. Cells were transfected with 5,000 plasmid genome equivalents or 25,000 plasmid genome equivalents per cell. The resulting impact on host replication fork elongation was monitored by (A) DNA Fiber Analysis and (C,D) the impact on host DDR signals using western blots. DFA data is represented as median of 2 independent biological replicates. The levels of REP 68/78 protein in pAAV2 transfected cells were monitored by western blot. (TIFF) [file ppat.1013142.s002.tiff]

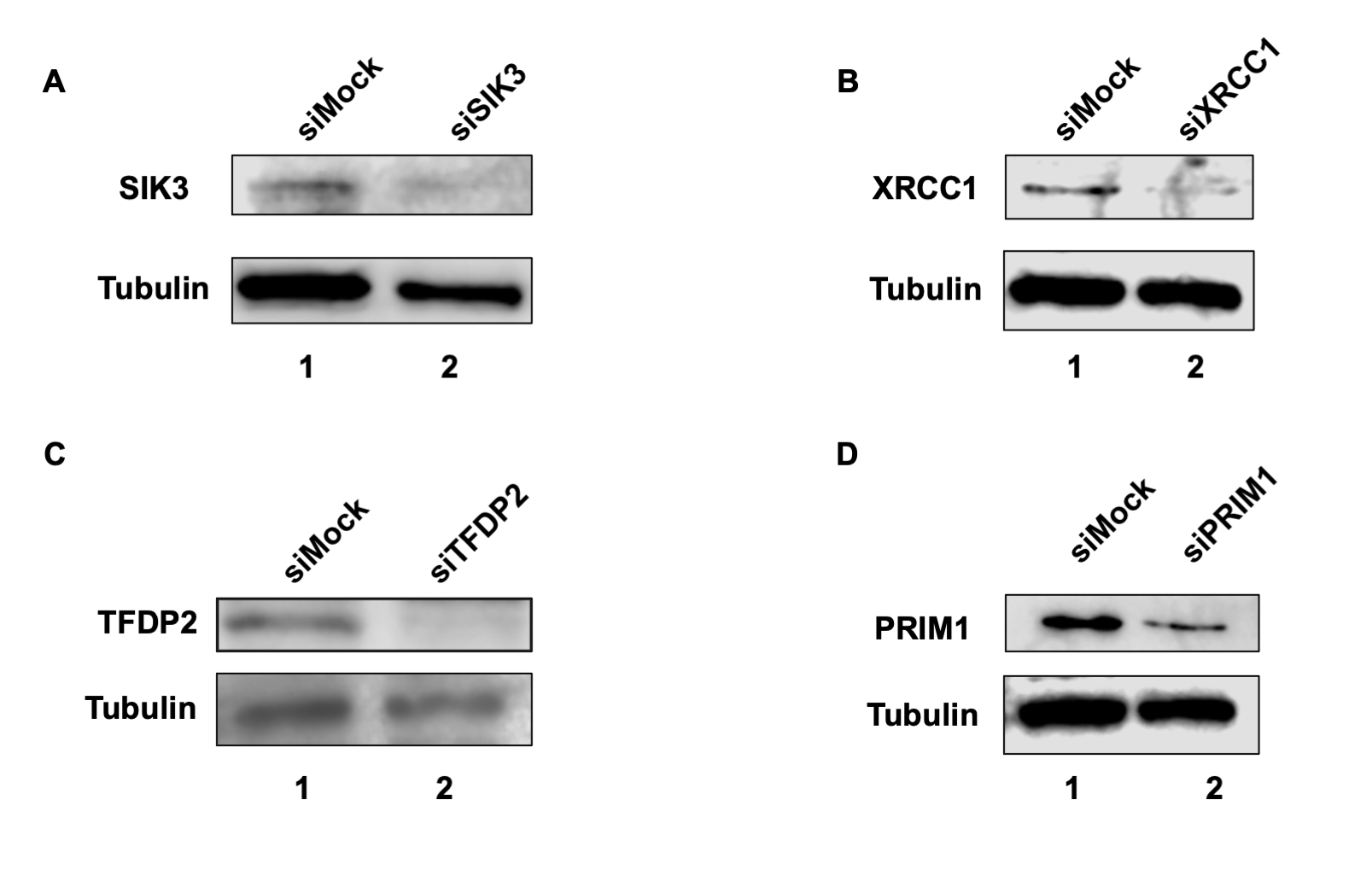

Supplement: S3 Fig — The knockdowns of (A) SIK3, (B) XRCC1, (C) TFDP2 and (D) PRIM1 in 293T cells were verified by western blots at 24 hours. (TIFF) [file ppat.1013142.s003.tiff]

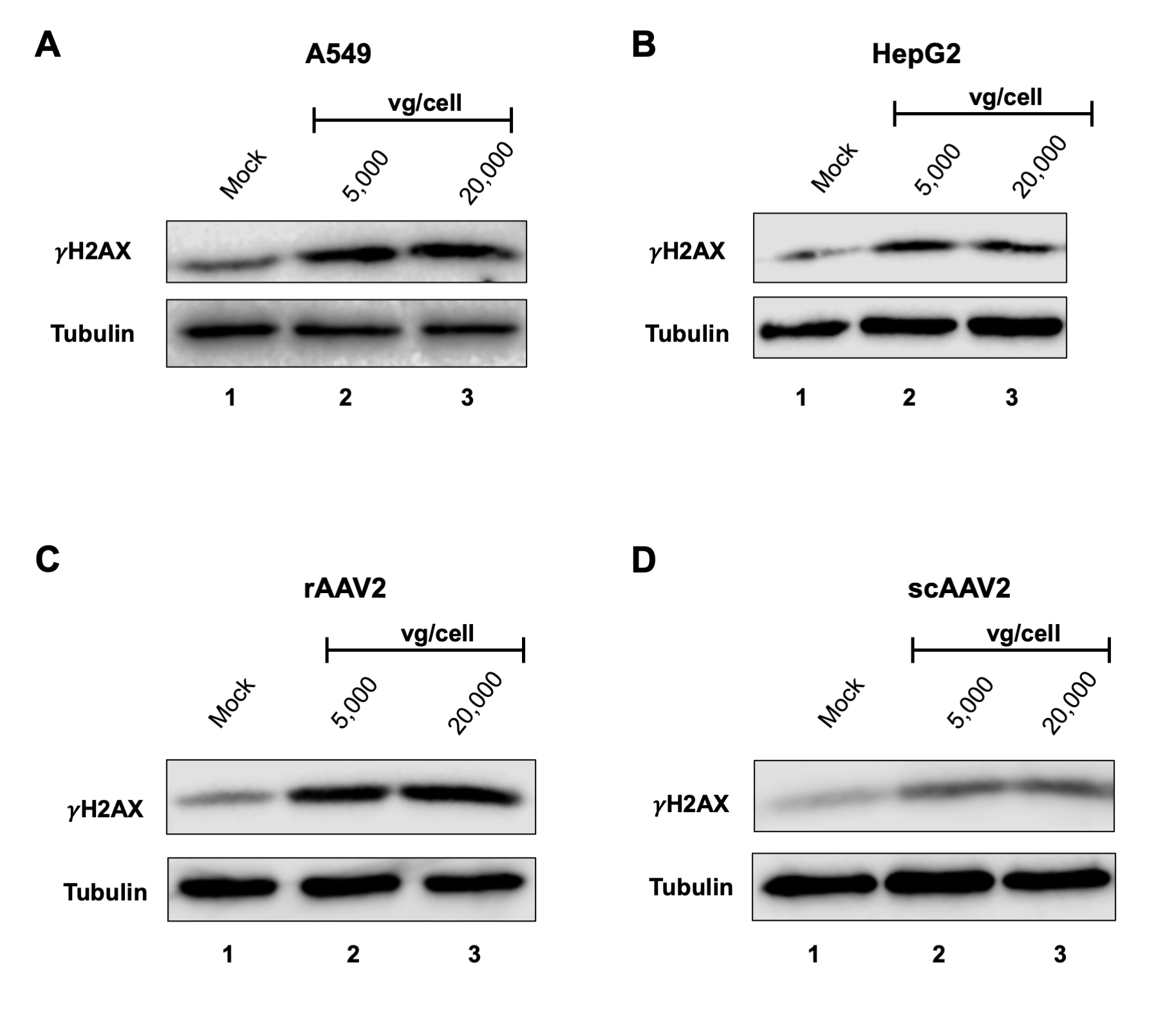

Supplement: S4 Fig — The impact on wtAAV2 infection on host genomes were monitored by gamma H2AX western blots upon infection of (A) A549 and (B) HepG2 cells with the indicated viral genomes per cell for 24 hours with tubulin levels as loading control. The impact of rAAV2 and scAAV2 transduction on host genome stability of 293T cells was monitored by gamma H2AX western blots using the indicated MOIs of (C) rAAV2 and (D) scAAV2 vectors. (TIFF) [file ppat.1013142.s004.tiff]

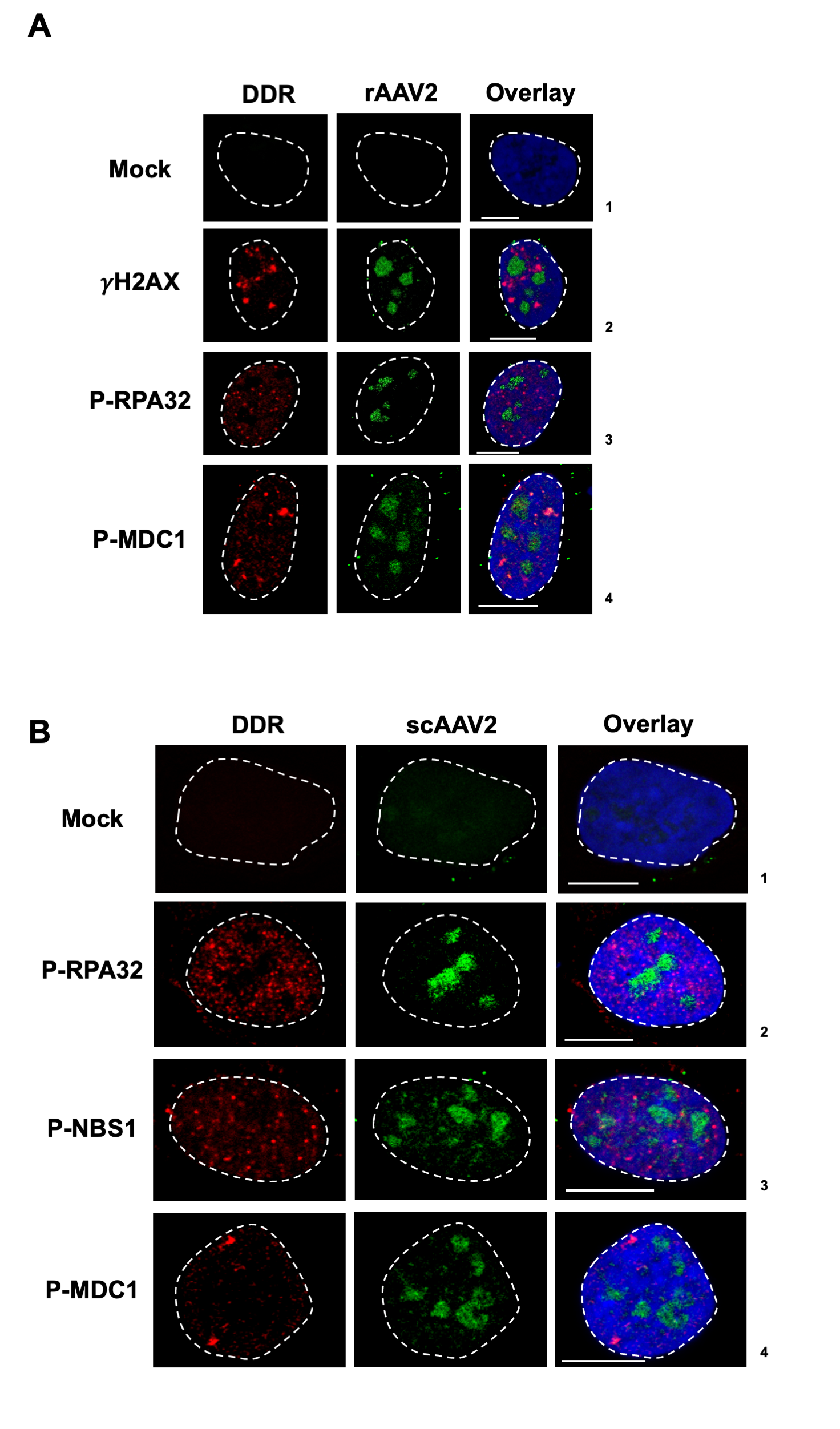

Supplement: S5 Fig — Immuno-FISH assays were performed to monitor to relative location of (A) rAAV2 and (B) scAAV2 genomes with that of the indicated phosphorylated DDR markers. DAPI staining was used to demarcate with nuclear borders with dashed lines and scale bars represent 10 microns. (TIFF) [file ppat.1013142.s005.tiff]
