# Supplementary material for: Adeno-Associated Virus 2 (AAV2) - Induced RPA exhaustion generates cellular DNA damage and restricts viral gene expression
Source: PLoS Pathog. 2025 Aug 18;21(8):e1013142. doi: 10.1371/journal.ppat.1013142 (PMC12373274; doi:10.1371/journal.ppat.1013142)
Supplement: Attachment — Submitted filename: Response to Reviewers.docx [file ppat.1013142.s007.docx]

**Response to Reviewers**

We thank the reviewers for their insightful and evaluative comments. In line with their suggestions, we have performed additional experiments to substantiate our findings in independent model systems of AAV infection. We have also extended our studies to rAAV and scAAV to evaluate the generalizability of our findings to the application of rAAV/scAAV gene therapy vectors in the clinic. These findings demonstrate the potential impact of our investigations in answering long-standing conundrums in the AAV/rAAV field. Our specific responses to the reviewer’s comments are described below (critiques in red and our responses in blue). The corresponding changes in the manuscript are highlighted in yellow in the marked-up document.

**Reviewer 1**

Major points:
1. Fig. 2 demonstrated that wtAAV2, rAAV2, and scAAV2 induce similar levels of replication stress. Fig. 5 showed that wtAAV2-mediated RPA32 exhaustion leads to cellular DNA damage. However, it is unclear whether rAAV2 can cause similar DNA damage, which could be explored by examining markers like rH2AX induction and colocalization of the AAV genome with DNA damage molecules (e.g., Fig. 5H). It is essential to test both single-stranded (rAAV) and double-stranded (scAAV) genomes, as they are used as viral vectors at high MOI. Since scAAV has a double-stranded genome, it may prevent RPA binding, which should be further examined.
**Response:** We thank the reviewer for this outstanding suggestion. We have performed additional experiments on rAAV/scAAV-mediated DNA damage, which are presented in Fig. S4C and S4D respectively (line 367-369). These findings show that both rAAV and scAAV induce cellular DNA damage detectable by induction of gamma H2AX at low MOI (lane 2), which is slightly elevated at higher MOIs (lane 3). These findings are independently corroborated by the induction of phosphorylated H2AX, phospho-RPA, phospho-MDC1 and phosphor-NBS1 in rAAV/scAAV transduced cells (shown at single cell level in Fig. S5A and S5B respectively) (line 374-375). Surprisingly however, these imaging assays revealed that the vector genomes did not occupy the same nuclear compartments as the cellular DDR markers, which corroborates our prior findings using high-throughput sequencing to monitor AAV/rAAV genome localization. These findings show that eventhough rAAV/scAAV genomes induce cellular DNA damage, their association with cellular DDR sites follow a distinct mechanism than that of the AAV virus. We surmise that AAV2’s Rep 68/78 proteins might play a contributing role of the colocalization of AAV with DDR sites.

2. Testing on additional cell types in Fig. 5 would strengthen the study's conclusions and expand its relevance to rAAV use in gene therapy applications.

**Response:** We have expanded our studies into A549 (Fig. S4A) and HepG2 (Fig. S4B) cells infected with AAV2, which are representative of tissue types that are targets for clinical gene therapy vectors (airway epithelium and hepatocytes respectively). Our studies show that increasing MOI’s of wtAAV2 genomes increase the cellular DNA damage measured by gamma H2AX levels. We have additionally expanded these studies into rAAV (Fig. S4C) and scAAV (Fig. S4D) transductions, discovering that both gene therapy vectors increase the cellular DNA damage signals (line 367-369). Lastly, we have expanded out DNA fiber analysis to A549 and U2OS cells, which showed that AAV infection decreases replication fork lengths on the host genome (Fig. S1; line 169-173). Thank you for the suggestion.

Minor points:
1. For better readability and consistency, AAV should be labeled as wtAAV2 throughout the manuscript.

**Response:** We have labelled AAV as wtAAV2 throughout the manuscript. Thank you for the suggestion.

2. Lines 168-170 contain a suggestion that is not well-supported by the data; this statement should be modified to align with the observed results.
**Response:** Thank you for the suggestion. We have removed this line from the revised manuscript to eliminate ambiguity.

3. In Fig. 3E, the manuscript should include data showing the knockdown efficiency of XRCC1, SIK, PRIM1, and TFDP2.

**Response:** We thank the reviewer for their suggestion. We have now provided western blots showing the knockdown efficiency of XRCC1, SIK3, PRIM1 and TFDP2 in Fig. S3. We have discussed these findings in the relevant section (line 275-276).

4. Lines 285-286 state, “plasmids expressing the RPA subunits (pRPA) into 293T cells prior to AAV2 infection as schematized in Fig. 4D.” The level of RPA expression should be confirmed and shown via Western blotting.

**Response:** We have provided the phospho-RPA overexpression western blot in Fig. 4I, lower half. Thank you for pointing this out.

5. Not sure if the iPOND MS original data need to be deposited in a database.

**Response:** Thank you. We have deposited the MS data to the MassIVE proteomics data repository and are available under the accession number MSV000098258.

**Reviewer 2**

Major points:

1. All the DNA fiber experiments and CHIP experiments were performed in HEK293 cells, whereas the replication fork experiments were performed in U2OS cells. Is there are a reason behind this that the authors want to articulate? This distinction is important since HEK293 cells express Adenoviral E1A which is a ssDNA binding protein involved in host factor recruitment. When taken together with T antigen, there may be effects that require further deconvoluting if there is involvement of such factors. An additional experiment or two to demonstrate E1A/T antigen do not alter the conclusions would be useful.

**Response:** We fully acknowledge that having adenovirus E1A and SV40 proteins might predispose 293T cells to more replication stress and subsequent DNA damage. We have therefore performed DFAs in non-virally transformed cell lines that are susceptible to AAV2 infection: U2OS and A549 cells (presented in Fig. S1) (line 169-173). These studies show that AAV2 infection leads to replication fork shortening in both cell types. However, it is important to note that all DFAs have an intrinsic heterogeneity caused by combining the replication forks from many cells. We therefore performed single-cell analysis using EdU-labelling of U2OS cells infected with AAV2 (presented in Fig. 1E) (line 173-183). The poor adherence of 293T cells to tissue culture plates precluded us from performing the EdU-labelled imaging of 293T cells. Taken together, these studies in U2OS, A549 and 293T cells support our overall model that AAV2 infection induces cellular replication stress monitored by DNA fiber analysis and this is independent of adenovirus E1A and SV40 Large T antigen. We thank the reviewer for their suggestion.

2. In Figure 2, the difference between rAAV and AAV2 is clear. Additional controls to the short ITR oligo would be transfection of AAV genomes without ITRs (from plasmid) and/or transfected extracted AAV genomes (from full particles, but without capsid).

**Response:** Thank you for the suggestion. We have performed these additional experiments and have presented them in Supplemental figure S2. We discover that heterologous pUC18 plasmid transfection did not induce replication stress (Fig. S2B) or cause cellular DNA damage (Fig. S2C), but the pAAV2 genomes induced both replication stress and cellular DDR signals in a dose-dependent manner (Fig. S2B and S2D respectively) (line 238-248). However, we point out that this might be caused by REP 68/78 expression from the plasmids. Taken together, these experiments reveal that non-specific double-stranded DNA molecules that are transfected into the host nucleus (at either low or high copy numbers) are not sufficient to cause cellular DNA damage or replication stress.

3. For Figure 4 and 5, were there additional experiments carried out with rAAV? While 4H shows some of this (protein read out), all AAV2 data is based on transcripts/epigenetic mods. The role of RPA2 here is also somewhat confusing here - as rAAV2 may not respond to RPA (Fig 4), but seems to have the same DNA damage response as AAV2 (Fig 2)?

**Response:** We have delved more deeply into the impact of rAAV/scAAV on the cellular DDR and vector gene expression. We have found that inhibition of RPA using the chemical inhibitor TDRL-505 leads to a de-repression of rAAV genome, leading to increased levels of GFP transcripts that are expressed (see modified Fig. 4H). We have further interrogated the dose-dependent impact of rAAV/scAAV on cellular DDR with western blots shown in Fig. S4C and S4D respectively (line 369-371). These studies revealed that both rAAV and scAAV induce cellular DNA damage in transduced cells, which is detectable at low MOI’s (5,000 vg/cell) and is increased at high MOI’s (20,000 vg/cell). Lastly, we have interrogated the relative location of rAAV/scAAV genomes to that of cellular DDR markers in the nucleus. Strikingly, we have found that rAAV/scAAV genomes do not colocalize with cellular DDR markers to the same extent as AAV genomes (Fig. S5A and S5B) (line 376-377). This suggests that rAAV and scAAV genomes might induce cellular DDR signals at a global level but not locally on the vector genome. These findings are worthy of substantial additional follow-up experiments that are beyond the scope of our current study.

Minor points:

1. The distinction between AAV2 vs rAAV2, AAV vs MVM, and viral vs host DNA damage is not always clearly articulated, when interpreting findings. Some careful delineation would be helpful for readers.
**Response:** Thank you for pointing this out. We have revised the manuscript to delineate the difference between the different genomes more distinctly.

2. The model in Figure 6 could be expanded to show how this affects AAV/rAAV gene expression and perhaps how this compares to other parvoviruses (such as MVM that this group has made significant contributions towards understanding in the past).

**Response:** Thank you for the suggestion. We have modified Figure 6 to incorporate MVM so that readers can compare the mechanisms of AAV versus MVM-induced DNA damage. We have provided this comparison in line 476-477.

**Reviewer 3**

Major points:

1. How specific is the damage induced by such high numbers of AAV2 genomes? Would transfecting the same amount of plasmid DNA also cause such reactions in the cell? A negative control of transfection 20,000 copies of plasmid DNA around the same size as the AAV2 genome would be a good comparison. In the same vein, is 20,000 copies of an AAV2 genome a physiologically relevant amount? This reviewer was somewhat disappointed that the lack of effect of rAAV2 was not further discussed since the introduction suggested that recombinant AAV2 vectors are important in the clinic, however, when no result was observed, further investigation was not carried out. It would be helpful to the reader to discuss the results in the context of how AAV is actually used in the clinic or infects cells, but these points are not discussed. Instead, the context of other viruses and the DDR is well described, perhaps even to the point where it seems less relevant to this study.
**Response:** We thank the reviewer for their comment and appreciate their concern about the specificity of our findings. We have, therefore, performed DNA fiber analysis and corresponding gamma H2AX western blots on 293T cells transfected with 5,000 and 25,000 genome equivalents of pUC18 plasmid and pAAV2 plasmid (presented in Fig. S2). We have found that compared with untransfected cells, the pUC18 plasmids are not sufficient to induce cellular replication stress (Fig. S2B) or cellular DDR (Fig. S2C). However, pAAV2 plasmids are sufficient to induce cellular replication stress (Fig. S2B) and dose-dependent DDR (Fig. S2D), likely caused by the expression of REP 68/78 proteins. This is likely because REP 68/78 has helicase and ATP-ase activity that interferes with the host genome non-specifically. We have also determined the impact of rAAV and scAAV genomes on cellular DDR signals using western blots, which show a dose-dependent induction of gamma H2AX in 293T cells at 24 hpi (Fig. S4C and S4D respectively) (line 238-248 and line 369-371). These findings are independently corroborated in U2OS cells by the induction of phosphorylated DDR markers upon transduction with rAAV and scAAV (Fig. S5A and S5B respectively) (line 376-377). Our exciting findings lend additional support to the importance of our model to rAAV and scAAV biology. While we do believe that these findings are relevant to understanding rAAV-induced toxicity, we have focused on the biology of AAV and parvovirus-induced DNA damage signaling in this manuscript. These findings have additional relevance for understanding how latent phases of viral life cycle might impact host replication stability, which is why we chose to compare and contrast our findings with that of other DNA viruses. Definitive conclusions of rAAV-induced replication stress induced by clinical gene therapy vectors would require extensive in-vivo studies in mice, patient-derived organoids and primary cells using clinical gene therapy vectors, which are beyond the scope of the current manuscript. Nevertheless, we completely respect the reviewer’s critique and hope the additional data that we have presented in Figs. S1-S5 will provide enough evidence that this is an important and generalizable phenomenon in gene therapy and DNA virology.

2. It would be good to include a positive control for RPA subunits in fig 4a, meaning a region of the host that they should be bound.
**Response:** Thank you for the suggestion. We have performed qPCR analysis for FRA3B, a fragile genomic region on the human genome that undergoes DNA break during AAV2 infection (previously identified by us in Boftsi et al., Human Molecular Genetics, 2022).

Minor points:
1. AAV2 monoinfection induces replication stress in the absence of any helper or other virus. scAAV2 and rAAV2 are sufficient to cause replication stress, but neither the core nor VP or ITR alone are. This suggests that it is the combination of these components working together that causes the replisome stress. Stating this would be helpful to the reader. For example, it is unclear why after showing these data, the authors then went to iPOND – the logic should be better stated.
**Response:** Thank you for the suggestion. We have clarified this issue in the relevant sections and provided a transition to why we pursued iPOND-MS (which was to determine the host factors that are enriched at replication stress sites). (line 287-292)

2. With respect to the iPOND data, it is also not well justified why the authors only followed up on RPA, which they already suspected was Why not follow up on anything else? Logic is a bit unclear since RPA2 was already suspected to be important, as was CSNK1.
**Response:** Thank you for your suggestion. The enrichment of RPA on the iPOND datasets was surprising because we have previously observed RPA binding to parvovirus genomes, which would deplete these molecules from host replication forks. This conundrum led us to delve deeper into how RPA might synergize with non-replicative AAV life cycles and AAV-induced replication stress. We have clarified this logic in the text. (line 287-292)

3. When RPA is bound to the AAV2 genome, the authors observe some repression because when RPA is not bound to the AAV2 genome, as per 4C, this results in greater gene expression, as per 4G (if these have the same copy numbers of genomes infected). However, this does not explain why the length of the replisomes is not different. Can the authors please explain this?
**Response:** This is an important question. We were struck by the observation that RPA inhibition did not have a synergistic effect with AAV2-induced replication stress. We interpret this to mean that additional redundant single-stranded DNA repair pathways are activated upon RPA inhibition (such as PARP-signaling) that help the cell to overcome AAV2-induced replication stress. However, these pathways are not active on the AAV2 genome, which would explain why RPA inhibition leads to derepression of AAV gene expression. This might be caused by RPA’s newly discovered role in interacting with host chromatin modifiers like G9a. We have clarified this issue in the text (line 310-311).

4. The idea of RPA exhaustion is interesting but needs to be better explained. Does inhibition lead to exhaustion? Can inhibition be reversed by removal of the inhibitor?
**Response:** RPA exhaustion refers to the competitive depletion of available RPA molecules by extrachromosomal parvovirus genomes. On the other hand, inhibition refers to the blocking of the active site on RPA32 by TDRL-505. The non-replicative AAV2 genomes serve as an ideal platform to interrogate the competitive inhibition by altering the MOI. This “titrating” effect of AAV2 genomes serving as a decoy for cellular RPA and its corresponding effect on host replication fork stability is tested in Fig. 5B/5C. TDRL-505 is a reversable inhibitor, and its removal can reverse the effect of RPA inhibition in cycling cells, but this would require cell passages, which would also deplete the host of RPA molecules. We have clarified what we mean by RPA exhaustion in the text (line 346-347).

5. In fig 4I vs 4J – does the transfection of pRPA in 4J lead to a decrease in length with CIdU compared to IdU? It appears that is lower but no significance is noted.
**Response:** The transfection of pRPA leads to a partial decrease in median lengths of the eukaryotic replication forks, but we do not make any biological conclusions from this. However, it is important to note that these studies are performed in non-synchronous cells transiently transfected with the pRPA plasmid, which likely introduces significant levels of heterogeneity in expression in the target cells. Excessive amounts of RPA molecules may interfere with the precise interactions of proteins needed to maintain replication fork fidelity, and are worthy of follow-up investigations in synchronous models of AAV infection. In these experiments, however, we have focused exclusively on the ability of RPA overexpression to rescue AAV2-induced replication fork shortening.

6. The logic of U2OS vs 293T cells is a bit unclear. 293T cells still express some adenovirus proteins albeit not the ones that would promote AAV2 infection, but why the authors switch from one cell type to the other is not well explained or justified.
**Response:** We acknowledge that 293T cells, by virtue of expressing adenovirus E1A and SV40 Large T antigen, can have a different impact on replication fork stability upon AAV infection. Therefore, we have performed these studies in U2OS cells and A549 cells (Fig. S2), demonstrating that the phenomenon of AAV2-induced replication stress is generalizable. We switched to U2OS cells for the EdU-pulsed imaging assays due to the low adherence of 293T cells to dishes. We have provided a better explanation for this in text (line 169-183).

7. The interpretation that the MRN proteins co-localize with AAV2 genomes does not mean necessarily mean this is where the replisome stress is occurring – the resolution of the imaging doesn’t give you that proximity. It is likely but these caveats should be noted. Again the concern of cell type changes is unclear.
**Response:** We fully agree with this interpretation that is raised by the reviewer. We have modified the text to state that the viral genomes and the DDR factors are in spatial proximity. We have previously discovered that AAV2 genomes associate with cellular sites of DNA damage. However, it is important to note that the DDR sites are not necessarily the same regions where stress is occurring (at least, we have not seen evidence of this so far) (line 375).

8. The introduction and conclusion seem somewhat long winded without addressing some key points that should be discussed. For example, the points above with respect to cell types and caveats of interpretation.
**Response:** Thank you for the suggestion. We have incorporated these caveats into our manuscript, focusing on the cell types that have been tested in our study (line 421-423).

9. It is not clear why inhibition of RPA phosphorylation would prevent its binding to the AAV2 genome? And even when it is not bound, the replisome length is not affected in 4E and 4F comparing AAV2 to AVV2+inhibitor there is no significant difference. This could be better explained.
**Response:** RPA binding to single-stranded DNA molecules lead to phosphorylation. TDRL-505 inhibits the DNA binding domain of RPA, thereby impeding its ability to associate with single-stranded DNA molecule. As a result, there is no phospho-RPA at this site either. We have discovered that all components of RPA associate with AAV and phospho-RPA32 is enriched on AAv2 genome, suggesting local DDR activation on the AAV2 genome. We have performed these ChIP assays using phospho-RPA32 antibodies as they are more potent at detecting DDR activation signals on viral genomes. The ChIP assay using TDRL-505 inhibitor is a confirmation that RPA activation is indeed inhibited on the AAV2 genome. As described earlier, it is likely that redundant single-stranded DNA repair pathways are activated by RPA inhibition (such as PARP-signaling) that help the cell to overcome AAV2-induced replication stress. This would explain why inhibitor+AAV2 does not enhance replication stress. We have explained the function of TDRL505 in the text (line 304, 453).

10. The authors switch between mono-infection or monoinfection

**Response:** Thank you. We have changed all instances of “monoinfection” to “mono-infection”.

11. Line 206-207 – typo with the word ‘be’
**Response:** We have taken out “be”. Thank you.

12. Line 310 – arising should be arise
**Response:** We have corrected this to “colocalize in the nucleus” to be more precise. Thank you.

13. Line 404 – underpinnings is misspelt

**Response:** We have corrected the spelling. Thank you.
